# Hypersensitive Reaction of Oxidized Regenerated Cellulose Powder in Total Knee Arthroplasty

**DOI:** 10.7759/cureus.59097

**Published:** 2024-04-26

**Authors:** Yoshinori Mikashima, Hitoshi Imamura, Yoshiko Shirakawa, Hiroshi Takagi, Ken Okazaki

**Affiliations:** 1 Oume Knee Surgery Center, Takagi Hospital, Tokyo, JPN; 2 Department of Orthopaedics, Tokyo Women’s Medical University Adachi Medical Center, Tokyo, JPN; 3 Department of Orthopaedics, Tokyo Women’s Medical University, Tokyo, JPN

**Keywords:** powder, lavage of product, hemostatic material, total knee arthroplasty, hypersensitivity, oxidized regenerated cellulose

## Abstract

The powdered form of oxidized regenerated cellulose (ORC powder) is a widely used biodegradable hemostatic material in the field of surgery. There are several reports of its effectiveness and safety; however, excessive foreign body reactions remain a concern for surgeons in total knee arthroplasty (TKA). A 70-year-old woman who underwent unilateral TKA using ORC powder to control perioperative blood loss exhibited a skin rash around her operated knee at six days postoperatively. These reactions were potentially hypersensitive to ORC powder. After receiving antiallergic medication for 18 days, the skin rash disappeared. Although there are several reports on the safety of ORC powder, inadequate intraoperative lavage of the product may induce hypersensitive reactions such as skin rash.

## Introduction

Perioperative blood loss is one of the major problems in total knee arthroplasty (TKA) [1]. Various techniques have been reported for controlling perioperative blood loss during TKA, such as combined intraoperative intravenous and intra-articular tranexamic acid administration [2], non-retention of suction drainage [3], use of drainage clamping with a suction tube [4], and non-use of a tourniquet [5]. Although combined intraoperative intravenous and intra-articular tranexamic acid administration has been reported as one of the best hemostatic treatments in TKA surgery, the average blood loss in their report was 686 ± 303 ml [2]. Therefore, further hemostatic treatments have been attempted to potentially reduce blood loss.

Powdered oxidized regenerated cellulose (ORC powder) is a widely used biodegradable hemostatic material [6]. In recent years, ORC powder has been used as an absorbable hemostatic agent in different clinical fields, including neurosurgery, thoracic surgery, general surgery, urologic surgery, and gynecologic surgery [7]. Although there is a report on the hemostatic effect and safety of the fiber form of ORC in TKA surgery, few studies have reported on its powder form [8]. The powder form of ORC remains a concern for its inability to remove excessive foreign bodies, while its fiber form is notably easy to remove after hemostatic use.

## Case presentation

A 70-year-old woman (weight, 68 kg; height, 147 cm; body mass index, 31.5 kg/m2) with a diagnosis of Kellgren-Lawrence grade 4 osteoarthritis underwent unilateral primary TKA (ATTUNE® cementless PS fixed bearing system; DePuy Synthes, Warsaw, IN, USA). The patient had a long history of well-controlled psychiatric illness. She had no history of diabetes (hemoglobin A1c; 5.5) and no hypoalbuminemia (albumin value before surgery; 3.9 g/dL). There was no history of drug use, metal allergy, or contact dermatitis.

The medial parapatellar approach and capsulotomy with patellar eversion were performed under a tourniquet pressure of 250 mmHg. The measured-resection technique was adopted for femoral and tibial bone resections. Identical PS fixed-bearing polyethylene inserts and cemented patellar resurfacing with an anatomically shaped patella were used. All components were implanted after pulsed lavage in high-pressure mode with 1000 cc saline (J Pulse®, TOKAI, Shizuoka, Japan). A periarticular injection of 40 mL of physiological saline solution containing 40 mg of triamcinolone acetonide was administered immediately before implantation. Then, the tourniquet was deflated, and hemostasis was achieved with an electrocoagulation knife. Two minutes after hemostasis, using 1 vial of SURGICEL® Powder (SURGICEL®-P; ETHICON, New Brunswick, NJ, USA), half of them were used for intra-articular tissues, and half of them were used for subcutaneous tissues. Pulsed lavage was reapplied in low-pressure mode with 1000 cc of saline. The capsule was subsequently sutured using STRATAFIX® (ETHICON, New Brunswick, NJ, USA), and wound closure was performed. The total operation time was 71 minutes. A tube drain was used for intra-articular drainage, and another was used for subcutaneous drainage. The former was clamped for 90 minutes with 1000 mg (10 mL) of tranexamic acid and 40 mL of physiological saline. Elastic stockings were used immediately after the surgery on the operated knee and before the surgery on the non-operated leg. A foot pump was used for 24 hours after surgery. The stockings were taken off four days postoperatively because she could gait smoothly. No anticoagulant was used. Range of motion exercises were started one day postoperatively, and gait exercises were started two days postoperatively. Continuous epidural anesthesia (150 ml 0.2% ropivacaine, 12 ml fentanyl, and 32 ml physiological saline) was administered for three days after surgery. Pain control by oral painkillers (celecoxib, 400 mg/day; tramadol, 200 mg/day; acetaminophen, 1600 mg/day) was also administered beginning one day postoperatively. Cefazolin (3g/day) was administered for three days after surgery. Then, Cefaclor (750mg/day) was administered for five days.

**Figure 1:**
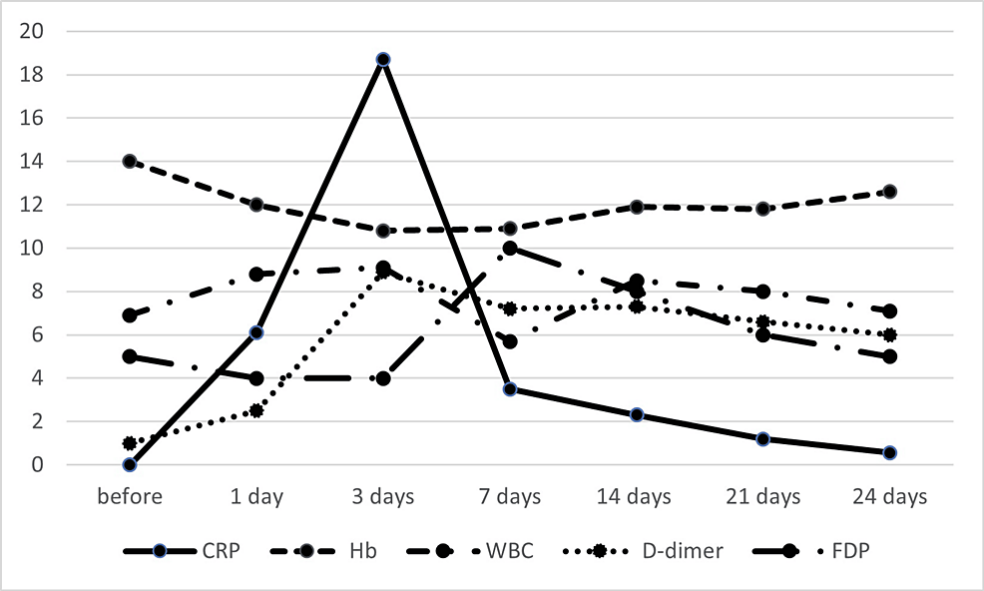
Transition of each blood marker postoperatively Line graphs show the postoperative changes in the levels of each blood marker. Hb (g/dl): hemoglobin; CRP (mg/dl): C-reactive protein (mg/l); WBC (103/μl): white blood cell count; FDP (μg/ml): fibrinogen/fibrin degradation product

The amount of drainage tube used 24 hours after surgery was 360 mL, and a postoperative decrease in the hemoglobin level was observed (Figure 1a). An acute increase in serum C-reactive protein (CRP) and a peak level of 18.3 mg/L (reference level 0-.3 mg/L) were observed three days postoperatively (Figure 1).

**Figure 2:**
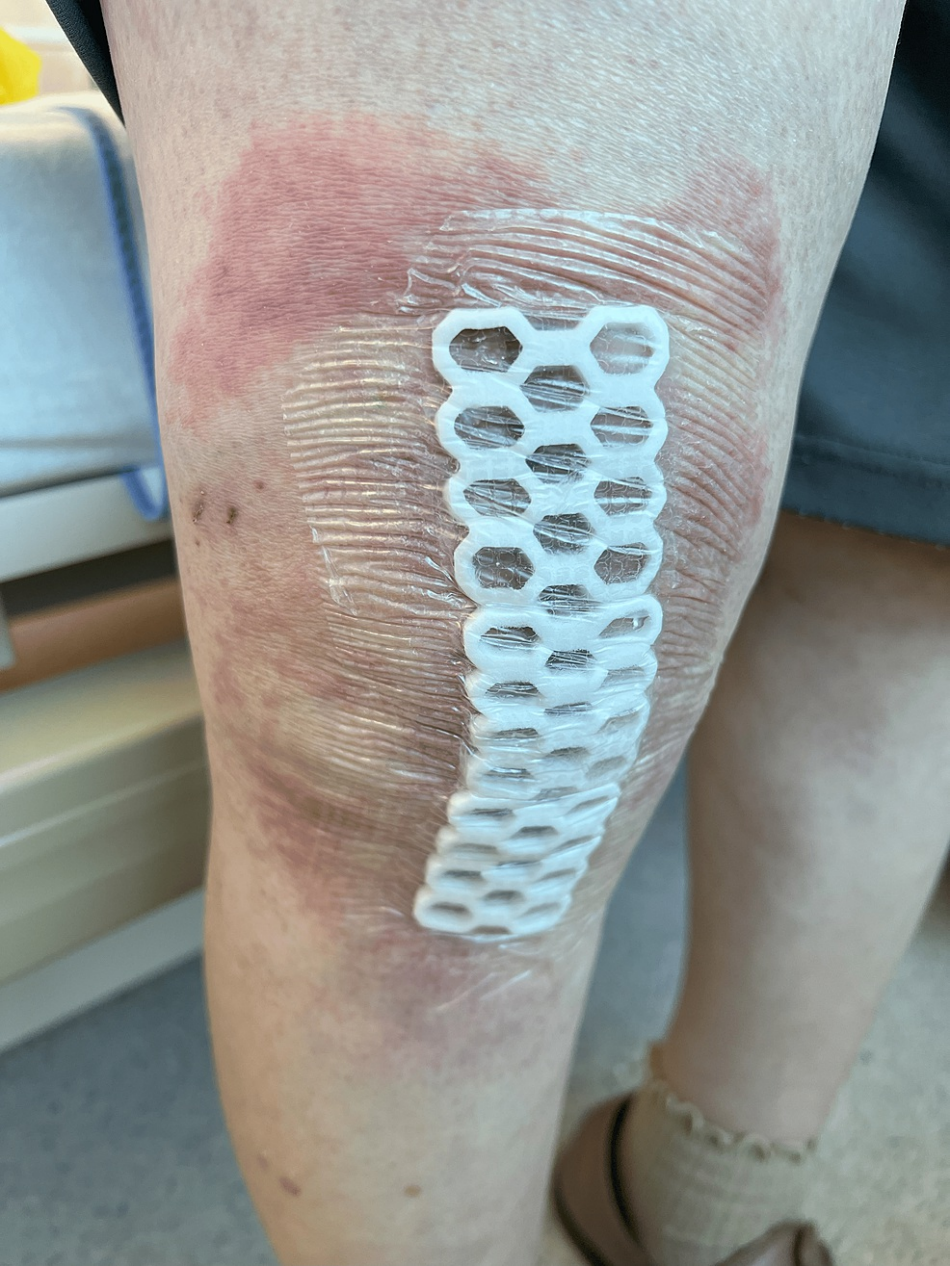
The knee was operated on six days postoperatively Skin rash around the operated knee appeared six days postoperatively.

There was no acute increase in the white blood cell count (WBC) or creatine phosphokinase (CPK) level. A skin rash around the operated knee appeared six days postoperatively (Figure 2).

**Figure 3:**
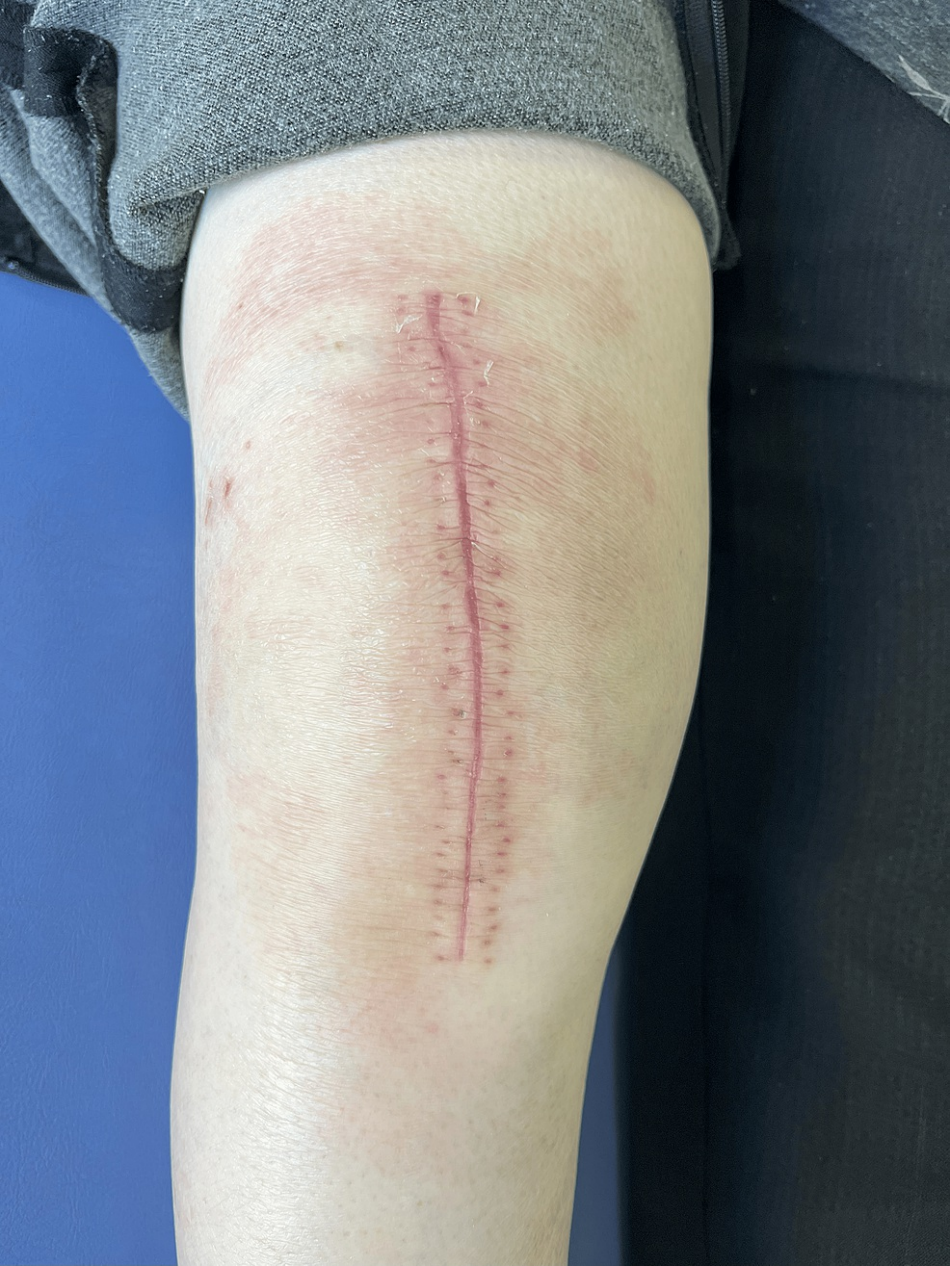
The operated knee 24 days postoperatively Skin rash around the operated knee nearly disappeared after 18 days of antiallergic medication.

Celecoxib administration was stopped immediately, and oral antiallergic medication (olopatadine) was started. The patient developed a fever up to 37.3°C four days postoperatively; however, she has not since exhibited a fever over 37.0°C. The three-day postoperative visual analog scale (VAS) score at rest was 3/10. When a skin rash appeared six days postoperatively, the VAS score remained at 3/10. Qualitative assessments, such as routine urine and microscopic evaluations, were normal. After 18 days of treatment with antiallergic medication, this skin rash nearly disappeared (Figure 3). The Hoffman sign was not positive at a regular check-up.

## Discussion

The most important finding of this report was that a hypersensitivity reaction to ORC powder was observed. A skin rash around the operated knee was observed postoperatively. We inferred a diagnosis per exclusionem that excessive ORC powder may have caused inflammatory foreign body reactions. It is important to eliminate other potential causes of skin rash around the operated knee, and six possible differentiations can be made. First, the possibility of a drug eruption of celecoxib was considered. However, many reports of celecoxib eruptions were not localized but rather generalized [9,10]. Second, the possibility of drug eruptions due to cefazolin and cefaclor was considered. However, reports of cefazolin eruptions have been attributed to photorecall and generalized effects [11]. Third, an intolerance to the dressing was considered. However, in this case, a skin rash was not observed in other dressing areas, such as fixing tapes of the urinary catheter and epidural tubes or tube drains from the operated knee. Fourth, metal allergies for implants and metal staplers were considered. However, the patient had no history of a metal allergy. Fifth, deep vein thrombosis was also considered. Deep vein thrombosis could not be ruled out because the peak values of D-dimer and FDP were 8.9 μg/ml and 10 μg/ml, respectively. However, considering the Hoffman sign was not positive throughout the regular check-up, a strong relationship between the skin rash and deep vein thrombosis was excluded. Sixth, hypersensitivity to sutures was considered [12]. Absorbent threads were used for the joint capsule and subcutaneous suture in this surgery. However, localized edema and redness of the wounds were not observed. Therefore, a strong relationship between the skin rash and hypersensitivity to sutures was excluded. Lastly, infection was also considered. Ishii et al. [13] reported that infected patients after TKA usually have a spike in fever within four weeks after surgery. No spike in fever, decreasing CRP, or normal qualitative urine evaluation were observed in this patient, which excluded the possibility of acute infection.

There are a few reports of inflammatory foreign body reactions with granuloma or pseudotumor formation created by ORC, particularly in cases exhibiting the presence of excess product [14,15]. Moreover, previous reports have described “mass effect” complications due to ORC swelling that resulted in neural compression syndromes [15,16]. Lin et al. [17] also reported severe pelvic abscess formation after laparoscopic myomectomy caused by intraoperative ORC placement. Al-Attar et al. [6] recommended irrigating or aspirating excess powder once hemostasis is achieved while simultaneously leaving the clot undisturbed.

ORC is a biodegradable, absorbable, and topical hemostatic agent. The properties and characteristics of oxidized cellulose were first reported by Yackel et al. [18] in 1942. According to their study, ORC is a plant-derived oxidized cellulose that demonstrates advantages in terms of safety, ease of operation, and clinical efficacy [19]. Currently, many surgical fields utilize ORC powder as an absorbable hemostatic material [7]. The material acts as a stable matrix for clot formation; furthermore, the material also enhances platelet activation and adhesion [6]. Moreover, ORC has been reported to produce antibacterial effects in a wound-healing animal model due to its creation of local acidic conditions [20].

Although the effectiveness and safety of ORC powder have been reported in various fields, there are no reports on the appropriate timing for the use of ORC powder in TKA, i.e., before or after implantation. In addition, there are no reported recommendations on the appropriate amount of powder and the way to wash excessive powder in TKA while disturbing the clot. Further studies are needed to resolve these issues. In this report, a case of a hypersensitive reaction to ORC powder was observed. Although there are several reports on the safety of ORC powder in various surgical fields, inadequate intraoperative lavage of the product may possibly induce hypersensitive reactions such as skin rash.

## Conclusions

This report described a hypersensitive reaction to ORC powder in a 70-year-old woman. Our results suggest that excessive use of ORC powder can potentially cause inflammatory foreign body reactions. Although there are several reports on the safety of ORC powder, the possibility of hypersensitivity reactions must be considered if a skin rash around the wound is observed after its use.
